# Development and Preliminary Validation of a Recombinant DP238L-Based Indirect ELISA for the Serological Detection of African Swine Fever Virus Antibodies

**DOI:** 10.3390/vetsci13101095

**Published:** 2026-10-15

**Authors:** Yuanfeng Zhang, Yong Xiao, Yongjie Jiang, Yongqi Wang, Jing Chen, Zhida Xiang, Chao Kang, Meilin Jin

**Affiliations:** 1 State Key Laboratory of Agricultural Microbiology, Huazhong Agricultural University, Wuhan 430070, China; zyfeng0821@webmail.hzau.edu.cn (Y.Z.); 924518497@webmail.hzau.edu.cn (Y.X.); jiangyongjie@webmail.hzau.edu.cn (Y.J.); wangyongqi123@webmail.hzau.edu.cn (Y.W.); 13997619420@163.com (J.C.); xzd@webmail.hzau.edu.cn (Z.X.); kangchaosuper@163.com (C.K.); 2 College of Veterinary Medicine, Huazhong Agricultural University, Wuhan 430070, China; 3 Hubei Jiangxia Laboratory, Wuhan 430200, China

**Keywords:** African swine fever virus, DP238L, indirect ELISA, recombinant antigen, serology, assay validation

## Abstract

African swine fever virus (ASFV) antibody detection is an important component of serological surveillance and retrospective investigations. DP238L was recently identified by proteome-wide serum-antibody profiling as a frequently recognized ASFV antigen, and recombinant DP238L was further shown to react with ASFV-positive sera and to elicit antigen-specific antibody responses in immunized pigs. Here, we extend those observations toward diagnostic assay development and preliminary validation. Full-length DP238L from ASFV strain SY-1 (GenBank OM161110) was evaluated as a single recombinant coating antigen for an indirect ELISA (DP238L-iELISA). Independent sequence analysis showed that SY-1 DP238L contains 238 amino acids, with a theoretical molecular mass of 27.13 kDa, a theoretical pI of 4.92, and a GRAVY value of −0.97. In a quality-restricted subset of complete, unambiguous 238-aa homologs (*n* = 7), amino acid identity to SY-1 ranged from 97.48% to 100% (median, 98.74%). Recombinant DP238L was expressed and purified from *Escherichia coli* and specifically recognized by ASFV-positive swine serum. ELISA optimization identified a coating antigen concentration of 0.5 μg/mL, a secondary-antibody working dilution of 1:2500 (0.4 μg/mL), a serum dilution of 1:100, a serum incubation time of 45 min, and a substrate development time of 15 min. Using 518 clinical swine serum samples categorized according to the commercial INgezim PPA COMPAC K3 blocking ELISA, ROC analysis yielded an AUC of 0.9989 (bootstrap 95% CI, 0.9973–0.9999). At the selected S/P cutoff of 0.30, positive percent agreement and negative percent agreement relative to INgezim were 97.2% and 98.7%, respectively; overall agreement was 98.3% and Cohen’s κ was 0.956. A limited six-sample heterologous panel provided preliminary cross-reactivity information, with all tested samples remaining below the cutoff. In a longitudinal panel from five experimentally infected pigs, three animals exceeded the cutoff at 7 days post-infection (dpi) and all five exceeded it by 10 dpi. These findings support DP238L as a promising recombinant antigen for ASFV serological assay development while emphasizing that the present results represent an internal comparator-based preliminary evaluation requiring broader independent validation.

## Simple Summary

African swine fever is a serious infectious disease of pigs, and antibody testing is important for surveillance and retrospective investigation. DP238L has recently been identified as an African swine fever virus protein that is strongly recognized by antibodies, but its performance as a diagnostic antigen has not been systematically evaluated. In this study, we produced recombinant DP238L and developed an indirect ELISA for antibody detection. The assay was evaluated using 518 swine serum samples and showed strong agreement with a commercial antibody test. In experimentally infected pigs, antibody responses crossed the assay cutoff in some animals by 7 days after infection and in all tested animals by 10 days. The assay also showed acceptable repeatability within the evaluated panel. These results extend previous evidence for the seroreactivity and immunogenicity of DP238L and support its further evaluation as a recombinant antigen for African swine fever serological testing.

## 1. Introduction

African swine fever (ASF) is a major infectious disease of domestic pigs and wild suids caused by African swine fever virus (ASFV), a large enveloped double-stranded DNA virus belonging to the family *Asfarviridae*. The ASFV genome is approximately 170–190 kb long and encodes more than 150 proteins involved in virion structure, replication, host interaction, and immune modulation [1,2,3,4]. Since the emergence of genotype II ASFV in China in 2018, both molecular and serological methods have remained important components of surveillance, outbreak investigation, and laboratory diagnosis [5,6].

ASFV serology has a long diagnostic history. Conventional ELISA and immunoblotting approaches were established using infected-cell antigens, followed by recombinant-antigen strategies designed to improve the specificity, standardization, and biosafety of antigen production [7,8,9,10,11,12]. Recombinant p30 and pp62 subsequently became well-characterized serodiagnostic targets, and comparative studies have demonstrated that antigen selection can substantially alter sensitivity, specificity, and confirmation requirements [13,14,15,16,17,18]. These findings remain directly relevant to the development of new ASFV antibody assays.

More recently, a wide range of ASFV recombinant proteins and assay formats have been evaluated, including pp62, pB602L, p22, p30, p54, p72, pK205R, I329L, p15, p17, p11.5, CD2v, and multiple antigen combinations [19,20,21,22,23,24,25,26,27,28,29,30,31]. Blocking, competitive, double-antigen sandwich, and nanobody-based assays have also been reported for p30, p54, p72, and K205R [32,33,34,35,36,37,38,39,40,41]. More recent work has continued to refine recombinant antigen production, multiepitope design, and combined indirect/blocking formats [42,43,44,45]. These studies highlight the importance of recombinant antigen selection, cutoff determination, and independent serum validation during ASFV ELISA development.

DP238L is a 238-amino-acid ASFV protein encoded in the right variable region of the viral genome. In 2026, Ma et al. used two ASFV antigen phage libraries and serum-antibody profiling to identify DP238L as a frequently recognized antigen, with major serum-reactive regions at residues 26–50 and 61–205 and variable positions at residues 62, 116, and 215 [46]. They subsequently expressed recombinant DP238L in *E. coli* and demonstrated its recognition by ASFV-positive swine sera by Western blotting and indirect ELISA. In addition, pigs immunized with recombinant DP238L developed strong antigen-specific antibody responses that were monitored by DP238L-coated indirect ELISA, supporting the antigenicity and immunogenicity of this protein [46]. Thus, Ma et al. established DP238L as a seroreactive and immunogenic ASFV antigen and highlighted its potential for serological applications. Their work, however, was centered on antigen discovery, epitope mapping, and immunogenicity rather than on development of a standardized diagnostic assay with an independently defined operating cutoff, large clinical-serum evaluation, comparison with an established commercial test, repeatability assessment, heterologous-serum testing, and infection-based seroconversion analysis using a fixed cutoff.

Accordingly, the present study addresses the next translational step by evaluating full-length DP238L as a single recombinant coating antigen for ASFV antibody detection and establishing an optimized indirect ELISA. Independent sequence and physicochemical analyses were performed using the SY-1 sequence and NCBI homologs, while only the positions of previously reported serum-reactive regions and variable sites were retained as literature annotations. Full-length recombinant DP238L was then expressed and evaluated for serum reactivity, followed by optimization of the DP238L-iELISA, S/P cutoff determination, preliminary cross-reactivity and endpoint-reactivity testing, intra- and interassay repeatability, method agreement with a commercial blocking ELISA in 518 serum samples, and evaluation of longitudinal antibody reactivity using sera from experimentally infected pigs.

## 2. Materials and Methods

### 2.1. Bioinformatic Analysis and Antigen Selection

The annotated DP238L coding sequence and translated protein sequence of the ASFV strain SY-1 were retrieved from the GenBank accession OM161110. The 238-amino-acid SY-1 DP238L protein was used as the reference sequence. Theoretical molecular weight, isoelectric point (pI), grand average of hydropathy (GRAVY), instability index, aliphatic index, aromaticity, and amino acid composition were calculated using Biopython (v1.86) [47]. Sequence hydropathy was calculated using the Kyte–Doolittle scale with a centered 19-residue sliding window [48].

DP238L homologous protein records were independently retrieved from the NCBI protein database in July 2026. Candidate records between 200 and 280 amino acids were retained, and exact duplicate amino acid sequences were collapsed, yielding 28 nonredundant DP238L-like records for quality assessment. Each retained sequence was globally aligned to the SY-1 reference using Biopython PairwiseAligner (match score, 2; mismatch score, −1; gap-opening score, −6; gap-extension score, −0.5). For each record, the protein length, number of ambiguous residues (X), and pairwise amino acid identity to SY-1 were recorded. Because the permissive 28-record set contained length-variable and partially ambiguous entries, a strict full-length subset was predefined as sequences that were exactly 238 aa long with no ambiguous X residues. This strict subset was used to summarize high-quality pairwise identity to the SY-1 reference.

**Figure 1:**
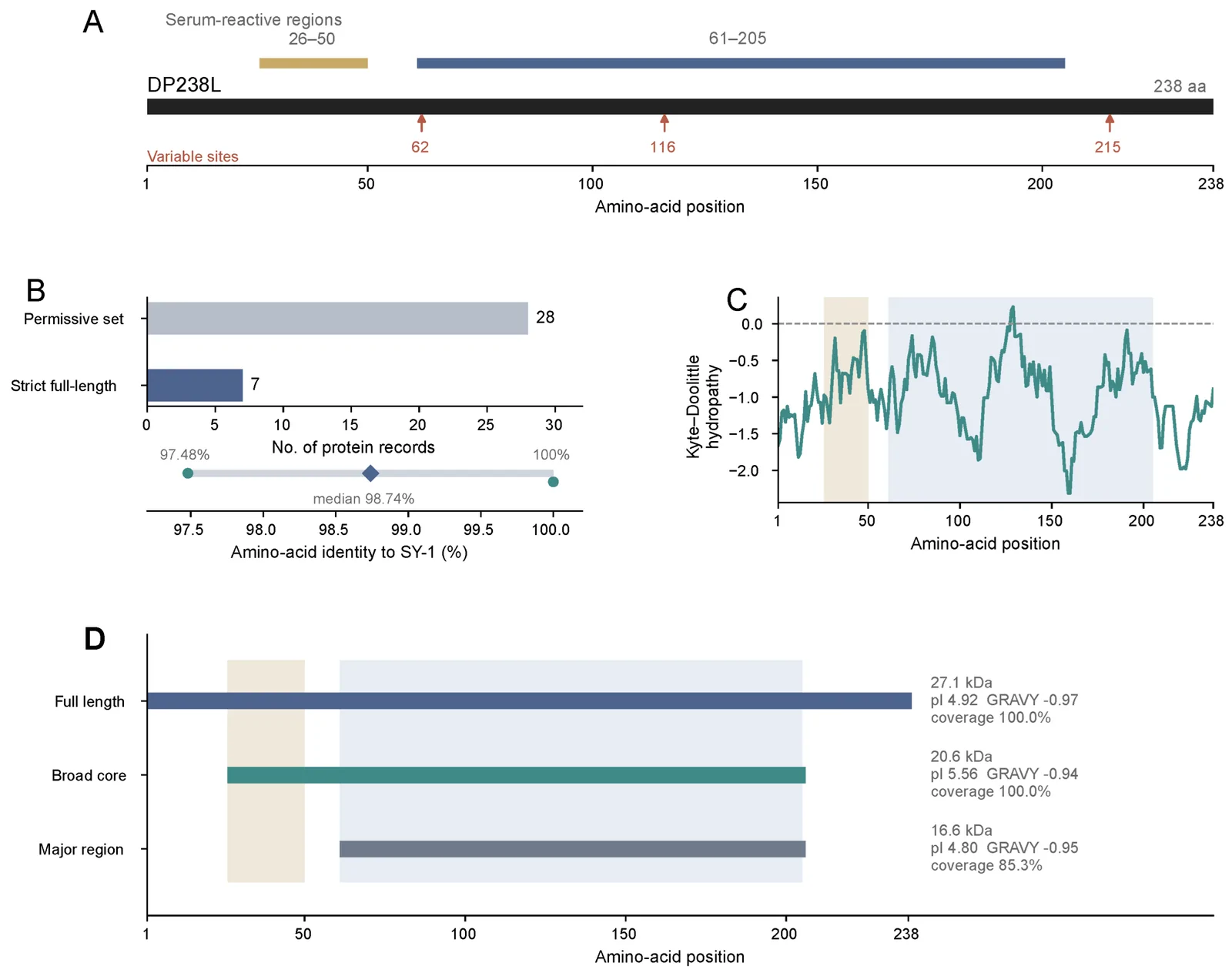
Independent sequence analysis and literature-annotated characterization of ASFV SY-1 DP238L for recombinant antigen selection. (**A**) Author-generated schematic of the 238-aa SY-1 DP238L reference sequence; the positions of the serum-reactive regions at aa 26–50 and 61–205 and variable residues 62, 116, and 215 are annotated from Ma et al. (2026) for biological context [46]. (**B**) Quality filtering of nonredundant DP238L-like protein records and amino acid identity of the strict full-length subset to SY-1; the permissive set contained 28 records, of which seven met the strict criteria of 238 aa with no ambiguous X residues, with identities ranging from 97.48% to 100% (median, 98.74%). (**C**) Kyte–Doolittle hydropathy profile of SY-1 DP238L calculated in the present study using a 19-aa sliding window. The teal curve represents the calculated hydropathy profile, and the gray dashed horizontal line indicates a hydropathy value of zero. Pale gold and pale blue shaded regions indicate the literature-reported serum-reactive regions at aa 26–50 and 61–205, respectively, for positional comparison. (**D**) Comparison of sequence coverage and physicochemical properties of the three candidate recombinant constructs. Blue, teal, and gray horizontal bars represent full-length DP238L (aa 1–238), the broad core (aa 26–205), and the major region (aa 61–205), respectively. Pale gold and pale blue shaded regions indicate the literature-reported serum-reactive regions at aa 26–50 and 61–205, respectively. Panels (**B**–**D**) were generated from analyses performed in the present study. No quantitative epitope-score or solvent-accessibility profiles from Ma et al. are reproduced.

Three recombinant-antigen designs were compared in silico: full-length DP238L (aa 1–238), a broad core spanning aa 26–205, and a major central region spanning aa 61–205. For each construct, the length, theoretical molecular weight, pI, and GRAVY were calculated. The previously reported serum-reactive regions at aa 26–50 and aa 61–205 and variable positions at residues 62, 116, and 215 were taken from Ma et al. and are shown only as positional literature annotations in the author-generated schematic in Figure 1A [46]. No quantitative epitope-score, conservation-profile, or solvent-accessibility plots from that publication are reproduced. SY-1 sequence analysis, homolog quality filtering and identity summary, Kyte–Doolittle hydropathy calculations, and recombinant construct comparisons were performed independently in the present study. The DP238L sequence from ASFV strain SY-1 was selected for recombinant expression because it was available from our previous work and corresponded to the challenge strain used in the longitudinal infection experiment.

### 2.2. Serum Samples and Study Design

A total of 518 archived clinical swine serum samples were collected for assay evaluation. Their serological status was not assigned independently before comparator testing. For the purposes of the present analysis, the 518 clinical sera were categorized solely according to the results obtained from the commercial INgezim PPA COMPAC K3 blocking ELISA (Eurofins Ingenasa, Madrid, Spain), yielding 376 comparator-negative and 142 comparator-positive samples. PCR results and epidemiological information were not used to define comparator-positive or comparator-negative status. These 518 sera were subsequently used to characterize the S/P distribution, generate the ROC curve, establish the operational cutoff, and evaluate method agreement. Comprehensive individual epidemiological metadata (including farm, geographic source, and animal category) were not consistently available for these archived field samples. Assay-condition optimization was performed using separate ASFV-positive and negative laboratory reference sera that were not counted among the 518 clinical samples. The repeatability, cross-reactivity, longitudinal, and endpoint-reactivity panels were evaluated separately, as summarized in Table 1.

Longitudinal serum samples were obtained from five experimentally infected pigs from a previously reported study [49]. Briefly, four-week-old Large White piglets were challenged orally with 500 HAD_50_ of genotype II ASFV SY-1 (GenBank OM161110) under ABSL-3 conditions; the strain identity and genotype have been described previously [49,50]. Infection in the original animal experiment was monitored by viral genomic DNA detection and hemadsorption assays as described previously [49]. All five animals included in the present longitudinal subset had serum available through 21 dpi. Serum samples available for the present study were collected at 0, 3, 7, 10, 14, and 21 days post-infection (dpi) and were used to evaluate longitudinal DP238L antibody reactivity (Table S3).

Six serum samples positive for non-ASFV swine pathogens/controls were used for the preliminary cross-reactivity assessment and included CSFV, PRV, PRRSV, PCV2, JEV, and CHO-vector control serum. An archived laboratory ASFV-positive reference serum previously confirmed as ASFV antibody-positive was used for endpoint-reactivity testing; an endpoint titer using the INgezim assay was not determined in the present study. Repeatability was assessed using 30 clinical sera (10 INgezim-positive and 20 INgezim-negative). Separate ASFV-positive and negative laboratory reference sera were used during assay-condition optimization. Internal reference/control sera were not counted as observations in the 518-serum ROC and method-agreement dataset.

All serum samples were stored at −20 °C until testing. The animal study protocols were approved by the Ethics Committee of Huazhong Agricultural University (Approval No. HZAUSW-2022-0030).

### 2.3. Expression and Purification of Recombinant DP238L

Unless otherwise specified, kanamycin, IPTG, Tris-HCl, NaCl, urea, imidazole, Coomassie brilliant blue, Tween-20, sodium fluoride (NaF), and chemicals used to prepare phosphate-buffered saline (PBS) and carbonate-bicarbonate coating buffer were obtained from Sinopharm Chemical Reagent Co., Ltd. (Shanghai, China).

The full-length DP238L coding sequence from ASFV SY-1 was synthesized by GenScript Biotech Corporation (Nanjing, China) and cloned into the pET-28a expression vector (Novagen, Madison, WI, USA) to generate an N-terminal His-tagged recombinant protein. The insert sequence and reading frame were confirmed by Sanger sequencing. The recombinant plasmid was subsequently transformed into *E. coli* BL21 (DE3) (TransGen Biotech Co., Ltd., Beijing, China). The cultures were subsequently grown in LB medium (Qingdao Hi-Tech Industrial Park Hope Bio-Technology Co., Ltd., Qingdao, China) supplemented with 50 μg/mL kanamycin to an OD_600_ of 0.6, induced with 1 mM IPTG, and incubated at 37 °C for 4 h. The cells were subsequently harvested and disrupted by sonication using a VCX600 ultrasonic processor (Sonics & Materials, Inc., Newtown, CT, USA) in lysis buffer (50 mM Tris-HCl, 300 mM NaCl; pH 8.0), after which the soluble and insoluble fractions were separated by centrifugation (Centrifuge 5425 R, Eppendorf, Hamburg, Germany).

The insoluble pellet containing inclusion bodies was solubilized in binding buffer (50 mM Tris-HCl, 300 mM NaCl, and 8 M urea; pH 8.0). The solubilized fraction was clarified by centrifugation and loaded onto a Ni-NTA resin column (GenScript Biotech Corporation, Nanjing, China) under denaturing conditions. On-column refolding was performed by washing the column with stepwise decreasing concentrations of urea (6 M, 4 M, 3 M, 2 M, 1 M, and 0 M) in wash buffer. The refolded His-tagged DP238L was then eluted with buffer containing 250 mM imidazole. The protein fractions were analyzed by SDS–PAGE and Coomassie brilliant blue staining, and the protein concentration was measured using a BCA protein assay kit (Sigma-Aldrich, St. Louis, MO, USA). The theoretical molecular mass of untagged SY-1 DP238L was 27.13 kDa; the expected mass of the final tagged construct was approximately 30 kDa.

### 2.4. Commercial ASFV Antibody ELISA Kit

The commercial comparator was the INgezim PPA COMPAC K3 blocking ELISA (Eurofins Ingenasa, Madrid, Spain), used according to the manufacturer’s instructions. The 518 clinical swine serum samples were tested using this assay, and the manufacturer’s blocking-percentage criteria were used to categorize samples as comparator-positive or comparator-negative. In the present study, the INgezim-defined classification was used as the comparator for ROC and method-agreement analyses and was not treated as an independent gold-standard reference.

### 2.5. SDS–PAGE and Western Blotting

Uninduced culture, induced culture, soluble fraction, insoluble fraction, and purified recombinant DP238L were separated by 12% SDS–PAGE using a Mini-PROTEAN Tetra vertical electrophoresis system (Bio-Rad Laboratories, Hercules, CA, USA). For Western blotting, proteins were transferred to PVDF membranes (Merck Millipore, Billerica, MA, USA) using a Mini Trans-Blot Electrophoretic Transfer Cell (Bio-Rad Laboratories, Hercules, CA, USA) and blocked with 5% skim milk. Recombinant protein identity was confirmed using an HRP-conjugated anti-His antibody (cat. no. ab202004; Abcam, Cambridge, UK; 1:5000). Antigenicity was evaluated using ASFV-positive swine serum as the primary antibody, followed by HRP-conjugated anti-swine IgG (cat. no. ab6915; Abcam, Cambridge, UK; 1:5000). A tag/vector control was included to distinguish DP238L-specific reactivity from reactivity against the fusion tag or bacterial contaminants. The signals were developed using an enhanced chemiluminescence (ECL) substrate (Advansta, San Jose, CA, USA) and recorded using a ChemiDoc XRS imaging system (Bio-Rad Laboratories, Hercules, CA, USA).

### 2.6. Establishment and Optimization of the DP238L-iELISA

The selected DP238L-iELISA working protocol was established as follows: High-binding 96-well microplates (cat. no. 3590; Corning Incorporated, Corning, NY, USA) were coated with 100 μL/well recombinant DP238L at a concentration of 0.5 μg/mL in 0.05 M carbonate-bicarbonate buffer (pH 9.6) overnight at 4 °C. The plates were washed three times with PBST (PBS containing 0.05% Tween-20) and blocked with 5% skim milk in PBST for 2 h at 37 °C; this blocking condition was selected during preliminary assay optimization. Swine serum was diluted 1:100 in PBST containing 1% skim milk, and 100 μL/well of diluted serum was added and incubated for 45 min at 37 °C. After three washes with PBST, the plates were incubated with 100 μL/well of HRP-conjugated anti-swine IgG (cat. no. ab6915; Abcam, Cambridge, UK) at a 1:2500 working dilution, corresponding to 0.4 μg/mL, for 45 min at 37 °C. Following five final washes with PBST, 100 μL/well of TMB substrate solution (Sigma-Aldrich, St. Louis, MO, USA) was added and incubated for 15 min at 37 °C in the dark. The reaction was stopped using 50 μL/well of 0.1 M NaF before absorbance measurement at 630 nm using a BioTek 800 TS absorbance microplate reader (model 800TS; BioTek Instruments, Winooski, VT, USA). This stopping procedure was part of our laboratory workflow; post-stop signal stability was not independently evaluated in the present study. Internal positive and negative controls were included on each plate for S/P normalization; however, independent quantitative plate-rejection criteria were not predefined.

During the optimization process, the concentrations of the coating antigen and secondary antibody were evaluated in a two-dimensional checkerboard matrix. DP238L coating concentrations of 0.25, 0.5, 1, 2, 4, and 8 μg/mL were tested against secondary antibody concentrations of 0.05, 0.1, 0.2, 0.4, 0.8, and 1.6 μg/mL. Positive and negative reference sera were tested under each condition, and the P/N ratio was calculated. Under the selected checkerboard conditions, serum dilutions of 1:50, 1:100, 1:200, and 1:400 were evaluated. Serum incubation times of 15, 30, 45, and 60 min and TMB substrate development times of 5, 10, 15, and 20 min were subsequently compared. Each plotted condition was represented by three replicate measurements, and mean P/N values with SDs were used for graphical presentation. Working conditions were selected considering the positive signal, negative background, and separation between positive and negative reference sera; the highest P/N value was not interpreted as evidence of statistical superiority.

### 2.7. Normalization and Cut-Off Determination

Each plate contained positive and negative internal controls. To reduce plate-to-plate variation, the primary normalized readout was the sample-to-positive ratio (S/P):

$$S/P=\frac{{\mathrm{OD}}_{\mathrm{sample}}-{\mathrm{OD}}_{\mathrm{NC}}}{{\mathrm{OD}}_{\mathrm{PC}}-{\mathrm{OD}}_{\mathrm{NC}}},$$

where ${\mathrm{OD}}_{\mathrm{sample}}$ is the mean sample ${\mathrm{OD}}_{630}$, and ${\mathrm{OD}}_{\mathrm{NC}}$ and ${\mathrm{OD}}_{\mathrm{PC}}$ are the mean negative and positive control ${\mathrm{OD}}_{630}$ values, respectively. Raw ${\mathrm{OD}}_{630}$ values were retained in the source dataset.

The 376 INgezim-negative samples were used to characterize the background S/P distribution and calculate a descriptive mean + 3SD benchmark. Because this distribution was right-skewed, the mean + 3SD value was treated as a descriptive reference rather than the sole basis for cutoff selection. ROC analysis was performed using all 518 serum samples with the INgezim-defined classification as the comparator. The threshold corresponding to the maximum Youden index was calculated and compared with the selected operational cutoff of S/P=0.30. The 0.30 threshold was retained as a conservative operational cutoff after considering the negative-serum distribution and the sensitivity/specificity trade-off. The same 518-serum cohort was subsequently used for the method-agreement analysis; therefore, this analysis represents an internal comparator-based evaluation rather than independent external validation.

### 2.8. Cross-Reactivity and Endpoint Reactivity Testing

Preliminary cross-reactivity was assessed using serum samples positive for six non-ASFV targets/controls (CSFV, PRV, PRRSV, PCV2, JEV, and CHO-vector control serum) together with ASFV-positive and negative controls. Each sample was tested in triplicate, and replicate and mean S/P values were evaluated relative to the fixed cutoff. This limited panel was intended to identify detectable cross-reactivity under the tested conditions rather than to estimate population-level diagnostic specificity (Table S4).

Endpoint reactivity was evaluated using an ASFV-positive sensitivity reference serum tested at dilutions of 1:20, 1:80, 1:320, and 1:640. Three replicate measurements are summarized as the mean ± SD. Because only the tested dilution series was available, the endpoint-reactivity result was reported as the highest tested dilution remaining above the fixed cutoff of 0.30 rather than as an exact endpoint titer.

### 2.9. Intra- and Inter-Assay Repeatability

Repeatability was assessed using 30 clinical serum samples comprising 10 INgezim-positive and 20 INgezim-negative samples. For intra-assay repeatability, each serum sample was tested in triplicate on the same plate. For interassay repeatability, the same panel was tested in three independent runs on different days using independently coated plates (batches 202001, 202002, and 202003). Major assay components, including the recombinant antigen preparation and detection reagents, were kept consistent across runs. For each run, replicate wells were first averaged; the interassay CV was then calculated across the three independent run means. The mean S/P, standard deviation, and coefficient of variation (CV = SD/mean × 100%) were calculated for each serum sample. Individual intra- and interassay CV values for the complete 30-serum panel are provided in Table S1. The panel spanned a range of positive and negative reactivities but did not deliberately enrich specimens immediately adjacent to the 0.30 cutoff.

### 2.10. Clinical Evaluation and Method Comparison

After the DP238L-iELISA cutoff had been fixed at S/P = 0.30, the 518 serum samples were classified by DP238L-iELISA and compared with the commercial INgezim blocking ELISA using a 2 × 2 contingency table. Positive percent agreement (PPA), negative percent agreement (NPA), overall agreement, and Cohen’s κ were reported to quantify method agreement. Discordant samples were examined individually using DP238L S/P values and INgezim blocking percentages (Table S2).

### 2.11. Statistical Analysis

Statistical analyses were performed using Python (v3.10), scikit-learn (v1.7.2), SciPy (v1.15.3), and statsmodels (v0.14.5). ROC curves and AUC values were calculated from the continuous S/P values using the INgezim-defined classification as the comparator. The 95% confidence interval for the AUC was estimated by nonparametric bootstrap resampling (4000 iterations; random seed = 42). The maximum-Youden threshold was identified from the ROC curve. The distribution of S/P values among the 376 INgezim-negative sera was summarized using the median, interquartile range, skewness, excess kurtosis, and 95th, 97.5th, and 99th percentiles. At the selected operational cutoff of 0.30, PPA, NPA, and overall agreement were calculated from the 2 × 2 contingency table; exact binomial 95% confidence intervals were used for these proportions. Cohen’s κ and its 95% confidence interval quantified agreement beyond chance. Because the same 518-serum cohort was used for cutoff evaluation and subsequent agreement analysis, the resulting estimates were interpreted as internal comparator-based performance measures rather than estimates against an independently defined diagnostic reference standard.

## 3. Results

### 3.1. Integrated Sequence Analysis Supports Selection of Full-Length SY-1 DP238L

The SY-1 DP238L protein comprises 238 amino acids and has a calculated molecular mass of 27.13 kDa, a theoretical pI of 4.92, and a GRAVY value of −0.974, consistent with an overall hydrophilic sequence profile. Independent NCBI retrieval followed by permissive length filtering and exact-sequence deduplication yielded 28 nonredundant DP238L-like records for quality assessment (Figure 1B). Seven records met the strict criteria of an exact 238-aa length and the absence of ambiguous residues; within this strict subset, amino acid identity to SY-1 ranged from 97.48% to 100%, with a median of 98.74%.

For biological context, the previously reported serum-reactive regions at aa 26–50 and 61–205 and variable positions at residues 62, 116, and 215 are indicated in an author-generated schematic in Figure 1A [46]. No published quantitative epitope-score, conservation-profile, or solvent-accessibility plots are reproduced in the revised figure. Independently, the Kyte–Doolittle hydropathy profile calculated in the present study showed no extended strongly hydrophobic segment (Figure 1C). Comparison of three candidate recombinant constructs showed that full-length DP238L remained compact (27.13 kDa), overall hydrophilic (GRAVY, −0.974), and retained both reported serum-reactive regions (Figure 1D). On the basis of this combined rationale, full-length SY-1 DP238L was selected for recombinant expression and assay development.

### 3.2. Recombinant DP238L Expression, Purification, and Serum Reactivity

**Figure 2:**
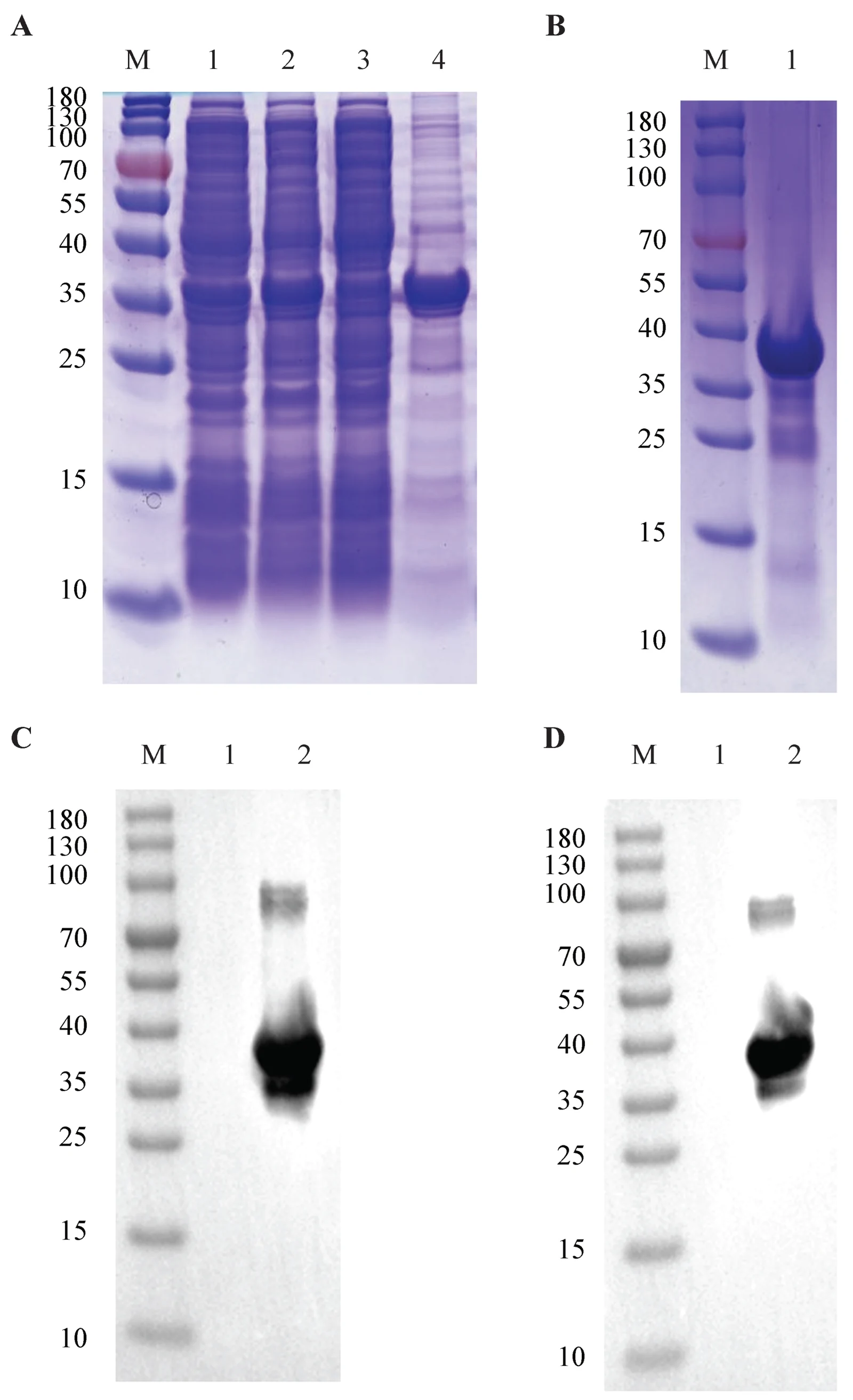
Expression, purification, and serum reactivity of recombinant DP238L. (**A**) Coomassie brilliant blue-stained SDS–PAGE analysis of recombinant DP238L expression and solubility. M, molecular-weight marker; lane 1, uninduced *E. coli* culture; lane 2, IPTG-induced culture; lane 3, soluble fraction; lane 4, insoluble fraction. (**B**) Coomassie brilliant blue-stained SDS–PAGE of purified and refolded DP238L recovered from the insoluble fraction. M, molecular-weight marker; lane 1, purified DP238L. (**C**) Western blot analysis using an anti-His antibody, with chemiluminescent detection. M, molecular-weight marker; lane 1, vector/tag control; lane 2, purified DP238L. (**D**) Western blot analysis using ASFV-positive swine serum, with chemiluminescent detection. M, molecular-weight marker; lane 1, vector/tag control; lane 2, purified DP238L.

Following IPTG induction, a prominent recombinant protein band was observed predominantly in the insoluble fraction, whereas the corresponding band was much weaker in the soluble fraction (Figure 2A). DP238L recovered from the insoluble fraction and subjected to refolding showed a dominant protein band after purification (Figure 2B). Western blotting with an anti-His antibody confirmed the identity of the recombinant DP238L protein, with a strong specific signal detected in the DP238L lane and no corresponding signal in the vector/tag control (Figure 2C). The purified recombinant protein was also specifically recognized by ASFV-positive swine serum, whereas the vector/tag control showed no detectable specific reactivity (Figure 2D). The calculated molecular mass of the His-tagged construct was approximately 30 kDa, whereas the recombinant protein showed an apparent electrophoretic mobility of approximately 35–40 kDa on SDS–PAGE; the basis for this anomalous migration was not further investigated. These results confirmed successful recombinant expression and purification of DP238L and demonstrated that the purified protein retained serological reactivity.

### 3.3. Optimization of the DP238L-iELISA

**Figure 3:**
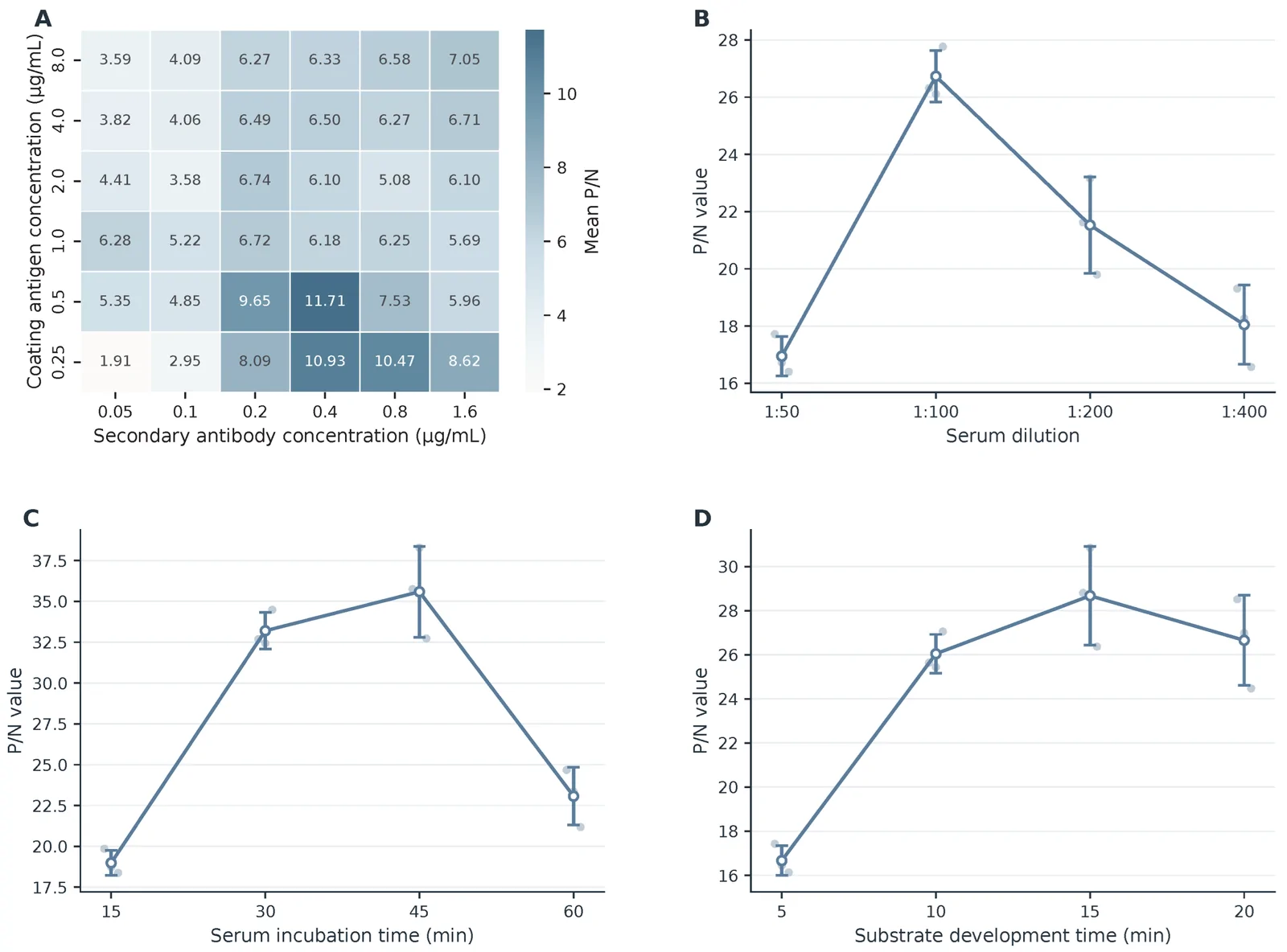
Optimization of the DP238L-iELISA. (**A**) Checkerboard matrix showing mean P/N values across coating-antigen and secondary-antibody concentrations. (**B**) Serum dilution optimization. (**C**) Serum incubation time optimization. (**D**) Substrate development-time optimization. In panels (**B**–**D**), pale points indicate replicate measurements, and the connected symbols show mean values with SDs.

Checkerboard analysis identified 0.5 μg/mL coating antigen and 0.4 μg/mL secondary antibody as the selected working combination, which also had the highest mean P/N value (11.71 ± 0.65; Figure 3A). Under the selected checkerboard conditions, a serum dilution of 1:100 produced the highest mean P/N value among the tested dilutions (26.73 ± 0.90; Figure 3B). The serum incubation experiment resulted in the highest mean P/N value at 45 min (35.59 ± 2.78; Figure 3C), and the substrate development experiment resulted in the highest mean P/N value at 15 min (28.68 ± 2.24; Figure 3D). These conditions were selected as working conditions for subsequent evaluation based on positive signal, negative background, and positive/negative separation; no statistical superiority among optimization conditions was inferred.

### 3.4. Cutoff Determination and ROC Performance

**Figure 4:**
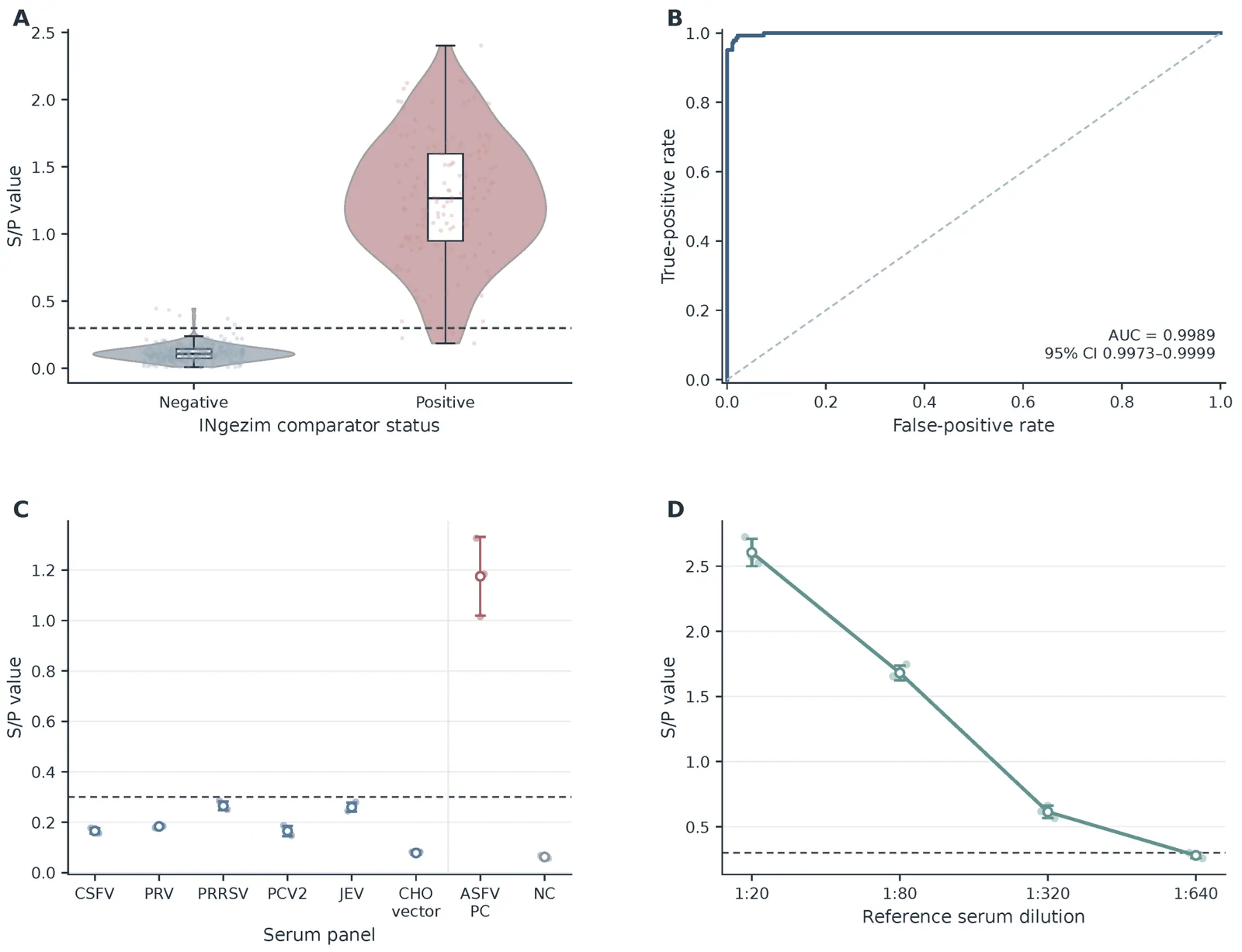
Threshold determination and analytical performance of the DP238L-iELISA. (**A**) Distribution of individual S/P values according to the INgezim-defined comparator status; the dashed horizontal line indicates the selected S/P cutoff of 0.30. (**B**) ROC curve for the 518-serum panel using the INgezim-defined classification as the comparator; the AUC and bootstrap 95% confidence interval are shown. The dashed diagonal line represents chance-level discrimination (true-positive rate = false-positive rate). (**C**) Preliminary cross-reactivity panel showing replicate and mean S/P values for the six non-ASFV samples together with the ASFV-positive and negative controls. The dashed horizontal line indicates the selected S/P cutoff of 0.30. (**D**) Endpoint reactivity of an ASFV-positive reference serum tested at 1:20, 1:80, 1:320, and 1:640; points show replicate measurements, and the line shows mean values with SDs. The dashed horizontal line indicates the selected S/P cutoff of 0.30. CSFV, classical swine fever virus; PRV, pseudorabies virus; PRRSV, porcine reproductive and respiratory syndrome virus; PCV2, porcine circovirus type 2; JEV, Japanese encephalitis virus; PC, positive control; NC, negative control.

The 376 INgezim-negative sera exhibited a right-skewed S/P distribution (skewness = 1.51; excess kurtosis = 6.04), with a median of 0.108, an interquartile range of 0.069, and 95th, 97.5th, and 99th percentiles of 0.198, 0.220, and 0.337, respectively. Because the negative S/P distribution was right-skewed, the mean + 3SD value (0.290) was treated as a descriptive benchmark rather than as the sole basis for cutoff selection. ROC analysis using the INgezim-defined classification as the comparator yielded an AUC of 0.9989 (bootstrap 95% CI, 0.9973–0.9999; Figure 4B). The maximum-Youden threshold was 0.225, corresponding to a sensitivity of 99.3% and a specificity of 97.9%. Compared with the maximum-Youden threshold, use of the selected operational cutoff of 0.30 reduced sensitivity from 99.3% to 97.2% while increasing specificity from 97.9% to 98.7%, thereby favoring a more conservative classification of comparator-negative sera.

### 3.5. Preliminary Cross-Reactivity Assessment and Endpoint Reactivity

Within the limited cross-reactivity panel, all six non-ASFV samples (CSFV, PRV, PRRSV, PCV2, JEV, and CHO-vector control serum) remained below the fixed S/P cutoff of 0.30 (Figure 4C; Table S4). Mean S/P values ranged from 0.078 to 0.265. The highest heterologous mean value (0.265) was only 0.035 below the cutoff and was therefore considered a borderline observation rather than evidence of comprehensive analytical specificity.

The S/P reactivity of the ASFV-positive reference serum progressively decreased with increasing dilution (Figure 4D). The highest tested dilution remaining above the cutoff was 1:320 (mean S/P = 0.614 ± 0.048), whereas the 1:640 dilution fell below the cutoff (mean S/P = 0.280 ± 0.021).

### 3.6. Repeatability of the DP238L-iELISA

Across the repeatability panel, the intra-assay CVs ranged from 0.00% to 9.35%, and the interassay CVs ranged from 0.00% to 10.83% (Table 2; Table S1). The classification of all 30 samples was unchanged across independent runs. The repeatability panel did not specifically enrich sera immediately adjacent to the 0.30 cutoff, which should be considered when interpreting repeatability around the decision threshold.

### 3.7. Concordance with Commercial Blocking ELISA

At the fixed S/P cutoff of 0.30, the DP238L-iELISA showed a positive percent agreement (PPA) of 97.2% (138/142; 95% CI, 92.9–99.2%) and a negative percent agreement (NPA) of 98.7% (371/376; 95% CI, 96.9–99.6%) relative to the commercial INgezim blocking ELISA. Overall agreement was 98.3% (509/518; 95% CI, 96.7–99.2%), with a Cohen’s κ of 0.956 (95% CI, 0.928–0.985), indicating high method agreement between the two assays under the present comparative framework (Table 3). The nine discordant samples comprised five DP238L-iELISA-positive/INgezim-negative and four DP238L-iELISA-negative/INgezim-positive sera; their individual S/P values and INgezim blocking percentages are provided in Table S2. No independent third-method adjudication was available for these discordant samples.

### 3.8. Longitudinal DP238L Antibody Reactivity in Experimentally Infected Pigs

**Figure 5:**
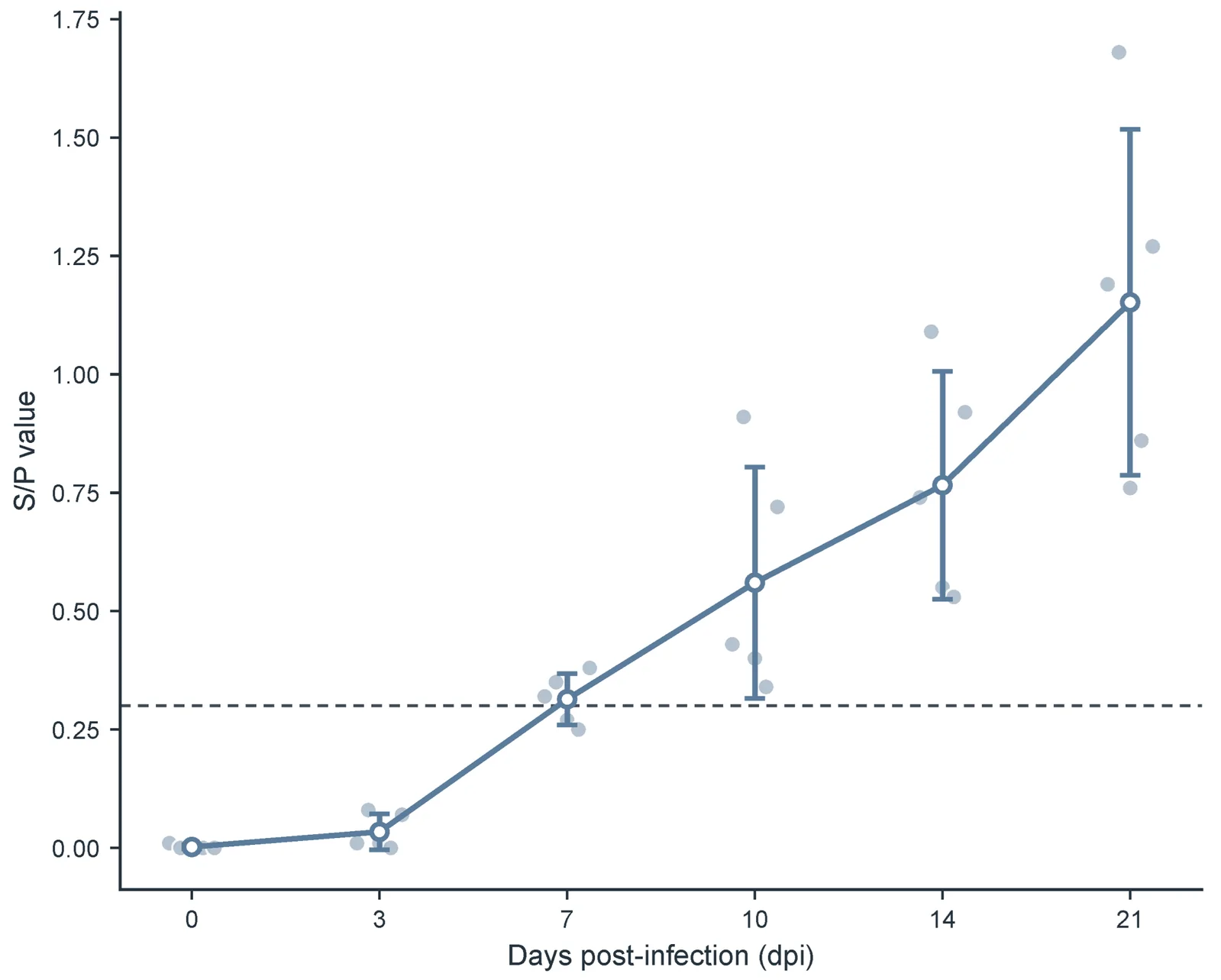
Antibody kinetics and seroconversion profile in ASFV-infected pigs. Longitudinal serum samples from five pigs (Pig-01 to Pig-05) were tested by DP238L-iELISA at 0, 3, 7, 10, 14, and 21 days post-infection (dpi). The dashed horizontal line indicates the fixed S/P cutoff of 0.30. Three pigs exceeded the cutoff at 7 dpi, and all five exceeded the cutoff by 10 dpi. Sampling days are shown at equally spaced positions on the x-axis.

Longitudinal DP238L antibody reactivity was assessed in serum samples from five experimentally infected pigs from 0 to 21 dpi (Figure 5; Table S3). At 0 and 3 dpi, all five animals remained below the 0.30 cutoff. At 7 dpi, three pigs exceeded the cutoff, with S/P values of 0.320, 0.349, and 0.376, whereas Pig-03 and Pig-04 remained below the cutoff with S/P values of 0.269 and 0.250, respectively. By 10 dpi, all five pigs exceeded the 0.30 cutoff (S/P range, 0.342–0.914), and S/P values continued to increase through 14 and 21 dpi. These observations provide preliminary evidence of early DP238L-specific seroreactivity following ASFV infection; however, the small cohort does not permit population-level inference regarding seroconversion timing.

## 4. Discussion

The present findings both confirm and extend the observations of Ma et al. [46]. In both studies, recombinant DP238L produced in *E. coli* was recognized by ASFV-positive swine sera, and DP238L functioned as a coating antigen in an indirect ELISA format. Ma et al. used proteome-wide antibody profiling and epitope mapping to identify DP238L as a widely recognized ASFV antigen and then validated its antigenicity by Western blotting and ELISA. Their subsequent immunization experiment showed that recombinant DP238L elicited strong and persistent antigen-specific antibody responses in pigs. Together with the present Western blot and ELISA data, these independent observations strengthen the evidence that DP238L contains antibody-accessible determinants that remain seroreactive after recombinant production.

There are also technical similarities and differences between the two DP238L ELISA applications. Both studies used high-binding plates, carbonate-bicarbonate coating buffer at pH 9.6, overnight coating at 4 °C, and a 1:100 serum dilution [46]. Ma et al. used 1 μg/mL recombinant antigen, a 1-h serum incubation, a 1:5000 HRP-conjugated anti-pig IgG dilution, a 10-min TMB reaction, and OD_450_ measurement. In the present study, checkerboard and stepwise optimization selected 0.5 μg/mL coating antigen, 0.4 μg/mL secondary antibody, a 45-min serum incubation, and a 15-min TMB reaction with OD_630_ measurement. These procedural differences should not be interpreted as evidence that one format is intrinsically superior; rather, they reflect independent optimization for different experimental purposes.

The principal distinction between the two studies lies in those purposes and in the validation framework. Ma et al. used DP238L-based ELISA mainly to confirm antigen-specific binding in ASFV-positive sera and to characterize antibody responses elicited by recombinant DP238L immunization, with three pigs per immunization group [46]. In contrast, the present study evaluated DP238L specifically as a diagnostic coating antigen. An operating S/P cutoff of 0.30 was established, and the assay was evaluated using 518 clinical swine serum samples, yielding an ROC AUC of 0.9989. The assay was also benchmarked against a commercial INgezim blocking ELISA, showing 98.3% overall agreement and a Cohen’s κ of 0.956, and was assessed for intra- and interassay repeatability and reactivity against a limited heterologous-serum panel. Accordingly, the present study extends the antigen-discovery and immunogenicity findings of Ma et al. into a standardized assay-development and preliminary internal-evaluation framework.

A further difference concerns the biological context of the longitudinal samples. Ma et al. monitored DP238L-specific antibody responses after immunization with recombinant DP238L, whereas the present study examined sera collected after experimental ASFV infection. In our longitudinal panel, three of five infected pigs crossed the fixed S/P cutoff by 7 dpi and all five were positive by 10 dpi (Figure 5). This infection-based seroconversion pattern complements the immunization-based kinetics reported by Ma et al. and is also consistent with the early serological responses described for established ASFV diagnostic antigens such as p30 and p54 [15,16]. Importantly, the two longitudinal experiments answer different questions: the previous study demonstrated the immunogenicity of DP238L itself, whereas the present study tests whether antibodies generated during ASFV infection can be detected by a fixed DP238L-iELISA operating procedure.

The clinical-serum results further support the potential utility of DP238L while remaining within the limits of a comparator-based study. The 376 INgezim-negative and 142 INgezim-positive sera showed strong separation of S/P values, with an ROC AUC of 0.9989 (Figure 4). At the selected cutoff of 0.30, comparison with INgezim yielded a PPA of 97.2%, an NPA of 98.7%, an overall agreement of 98.3%, and a Cohen’s κ of 0.956 (Table 3). Because the same INgezim-defined classification was used for ROC analysis and method comparison, these values should be interpreted as internal comparator-based performance rather than estimates against an independently defined diagnostic reference standard.

The preliminary cross-reactivity panel showed that all six non-ASFV samples remained below the fixed cutoff, whereas the ASFV-positive control was reactive (Figure 4C; Table S4). However, the highest heterologous mean S/P value was 0.265, only 0.035 below the cutoff. Given the single-sample representation of each non-ASFV target, this borderline observation warrants caution in field interpretation and does not support definition of a formal equivocal interval; borderline samples may benefit from confirmatory retesting. Endpoint testing showed that the positive reference serum remained above the cutoff at 1:320 but fell below it at 1:640 (Figure 4D), so 1:320 is reported only as the highest tested positive dilution rather than an exact endpoint titer. The repeatability analysis further showed intra-assay CVs of 0.00–9.35% and interassay CVs of 0.00–10.83% with stable classification of all 30 samples (Table 2), consistent with the repeatability framework commonly used for ASFV recombinant-antigen ELISAs [13,19,20,21,22,34].

Several limitations of this preliminary evaluation should be noted. First, because the INgezim assay was used to define the comparator classification for the 518 clinical sera, the ROC analysis and agreement estimates represent an internal comparator-based evaluation rather than validation against an independently defined reference standard. In addition, the same 518-serum cohort was used for ROC-based cutoff evaluation and subsequent agreement analysis, which may result in optimistic performance estimates. Although samples were tested according to predefined procedures, formal blinding between DP238L-iELISA and INgezim testing was not implemented. Comprehensive epidemiological metadata were not consistently available for the archived clinical sera, limiting assessment of geographic, herd-level, and age-category representativeness. The heterologous cross-reactivity panel was also limited to one sample per target; therefore, the S/P value of 0.265 observed near the cutoff should be interpreted cautiously and the present dataset is insufficient to establish a formal equivocal interval. The repeatability assessment was limited to 30 sera and did not deliberately enrich specimens immediately adjacent to the 0.30 cutoff; therefore, repeatability across broader serum panels and particularly around the decision threshold requires further evaluation. Regarding antigen characterization, the apparent molecular mass was not independently verified by mass spectrometry, Western blotting under denaturing conditions does not demonstrate complete restoration of conformational epitopes after refolding, and formal densitometric purity, residual urea/imidazole, endotoxin, storage stability, and antigen lot-to-lot reproducibility were not independently assessed. Finally, the longitudinal observations were derived from only five experimentally infected pigs and require verification in larger cohorts. As a methodological example, Abousenna et al. evaluated a lateral-flow antigen assay for canine parvovirus using live attenuated vaccine batches, comparing its performance with qPCR and reporting relative sensitivity, specificity, and accuracy [51]. This example illustrates the value of reference-method comparisons in veterinary assay evaluation. Prospective external and interlaboratory validation using independently characterized serum panels will be necessary before routine diagnostic application of the DP238L-iELISA.

## 5. Conclusions

Ma et al. established DP238L as a broadly recognized ASFV antigen and demonstrated its seroreactivity and immunogenicity using recombinant protein, indirect ELISA, and pig immunization [46]. The present study extends that work toward assay development. Full-length recombinant DP238L was incorporated into an indirect ELISA with a selected S/P cutoff of 0.30 and evaluated using 518 clinical swine sera categorized by a commercial INgezim blocking ELISA. The internal comparator-based evaluation yielded an ROC AUC of 0.9989, a PPA of 97.2%, an NPA of 98.7%, an overall agreement of 98.3%, and a Cohen’s κ of 0.956. Repeatability, preliminary cross-reactivity, endpoint reactivity, and longitudinal responses in five experimentally infected pigs were also characterized. These findings support further evaluation of DP238L as a recombinant antigen for ASFV serological assay development; broader independent, geographically diverse, and interlaboratory validation remains necessary before routine diagnostic use.

## Figures and Tables

**Table 1:** Serum panels and their roles in the assay-development and preliminary-evaluation workflow.

Panel	*n*	Source/Comparator Categorization	Purpose
Clinical serum panel	518	Serological status unknown at collection; subsequently categorized solely by the INgezim comparator as 376 comparator-negative and 142 comparator-positive samples	S/P distribution, ROC/cutoff evaluation, and method agreement
Longitudinal serum panel	5 pigs	Experimentally infected pigs sampled from 0–21 dpi	Longitudinal DP238L antibody reactivity
Non-ASFV cross-reactivity panel	6	Independent heterologous panel: CSFV, PRV, PRRSV, PCV2, JEV, and CHO-vector control	Preliminary cross-reactivity assessment
ASFV-positive reference serum	1	Archived laboratory reference serum previously confirmed as ASFV antibody-positive	Endpoint reactivity
Repeatability panel	30	10 INgezim-positive and 20 INgezim-negative clinical sera	Intra- and interassay repeatability

**Table 2:** Summary of intra- and interassay repeatability for the 30-serum panel.

Repeatability Measure	Intra-Assay	Interassay
No. of sera evaluated	30	30
Observed CV range (%)	0.00–9.35	0.00–10.83
Classification stability	30/30 unchanged	30/30 unchanged

**Table 3:** Method agreement between the developed DP238L-iELISA and the commercial INgezim blocking ELISA in 518 clinical swine serum samples.

	Blocking Immunoenzymatic Assay (INgezim)		
**DP238L-iELISA**	**Positive (+)**	**Negative (−)**	**Total (** ***n*** **)**
Positive (+)	138	5	143
Negative (−)	4	371	375
Total (*n*)	142	376	518

PPA = 97.2% (138/142); NPA = 98.7% (371/376); overall agreement = 98.3% (509/518); Cohen’s κ = 0.956.

## Data Availability

The anonymized sample-level data supporting the clinical-panel analyses are provided as Supplementary Data S1. Additional source data and analysis details are available from the corresponding author on reasonable request.

## Supplementary Materials

The following supporting information can be downloaded at: https://www.mdpi.com/article/10.3390/vetsci13101095/s1, The supporting information includes Table S1 (individual repeatability CV values); Table S2 (discordant sample analysis); Table S3 (individual longitudinal S/P values); Table S4 (preliminary cross-reactivity panel); and Data S1 (anonymized sample-level S/P and INgezim comparator data for the 518-serum clinical panel).

## Abbreviations

The following abbreviations are used in this manuscript:

ASF	African swine fever
ASFV	African swine fever virus
iELISA	Indirect enzyme-linked immunosorbent assay
S/P	Sample-to-positive ratio
P/N	Positive-to-negative ratio
ROC	Receiver operating characteristic
AUC	Area under the ROC curve
CV	Coefficient of variation
PPA	Positive percent agreement
NPA	Negative percent agreement
