# Supplementary material for: Development and Preliminary Validation of a Recombinant DP238L-Based Indirect ELISA for the Serological Detection of African Swine Fever Virus Antibodies
Source: Vet Sci. 2026 Oct 15;13(10):1095. doi: 10.3390/vetsci13101095 (PMC13709942; doi:10.3390/vetsci13101095)
Supplement: Supplementary file 1 [file vetsci-13-01095-s001.zip › Supplementary_Materials.pdf]

# Supplementary Materials

*Development and Preliminary Validation of a Recombinant DP238L-based Indirect ELISA for the Serological Detection of African Swine Fever Virus Antibodies*

## Table S1. Individual intra- and inter-assay CV values for the 30 sera evaluated for repeatability.

| Sample ID | INgezim Classification | Mean S/P Ratio | Intra-assay CV (%) | Inter-assay CV (%) |
|---|---|---|---|---|
| P-01 | Positive | 1.845 | 3.21 | 5.40 |
| P-02 | Positive | 1.420 | 0.00 | 4.12 |
| P-03 | Positive | 0.985 | 2.85 | 0.00 |
| P-04 | Positive | 2.110 | 3.52 | 5.91 |
| P-05 | Positive | 0.745 | 5.20 | 7.84 |
| P-06 | Positive | 1.150 | 4.05 | 6.55 |
| P-07 | Positive | 0.520 | 5.82 | 8.16 |
| P-08 | Positive | 2.340 | 2.91 | 4.38 |
| P-09 | Positive | 1.675 | 3.74 | 5.52 |
| P-10 | Positive | 0.890 | 4.41 | 7.15 |
| N-01 | Negative | 0.125 | 6.12 | 9.41 |
| N-02 | Negative | 0.085 | 5.54 | 8.85 |
| N-03 | Negative | 0.140 | 7.21 | 10.52 |
| N-04 | Negative | 0.095 | 4.86 | 7.64 |
| N-05 | Negative | 0.210 | 9.35 | 10.12 |
| N-06 | Negative | 0.115 | 5.92 | 8.24 |
| N-07 | Negative | 0.065 | 8.15 | 10.83 |
| N-08 | Negative | 0.175 | 4.63 | 7.41 |
| N-09 | Negative | 0.105 | 6.31 | 9.75 |
| N-10 | Negative | 0.090 | 5.28 | 8.52 |
| N-11 | Negative | 0.243 | 7.05 | 10.15 |
| N-12 | Negative | 0.130 | 5.76 | 8.92 |
| N-13 | Negative | 0.075 | 7.82 | 10.61 |
| N-14 | Negative | 0.155 | 4.95 | 7.25 |
| N-15 | Negative | 0.190 | 6.42 | 9.88 |
| N-16 | Negative | 0.080 | 8.41 | 10.22 |
| N-17 | Negative | 0.110 | 5.15 | 8.04 |
| N-18 | Negative | 0.225 | 6.84 | 10.45 |
| N-19 | Negative | 0.145 | 5.32 | 7.91 |
| N-20 | Negative | 0.070 | 8.71 | 10.54 |

The repeatability panel contained 10 INgezim-positive and 20 INgezim-negative sera. Samples immediately adjacent to the 0.30 cutoff were not deliberately enriched.

## Table S2. Analysis of discordant samples between the DP238L-iELISA and the commercial INgezim PPA COMPAC K3 ELISA.

| Sample ID | DP238L S/P Ratio | DP238L Result (cutoff=0.30) | INgezim Blocking (%) | INgezim Result |
|---|---|---|---|---|
| **P016** | 0.266 | Negative | 54.2 | Positive |
| **P019** | 0.230 | Negative | 61.5 | Positive |
| **P132** | 0.225 | Negative | 51.8 | Positive |
| **P009** | 0.186 | Negative | 73.4 | Positive |
| **N097** | 0.327 | Positive | 38.5 | Negative |
| **N105** | 0.369 | Positive | 24.2 | Negative |
| **N225** | 0.391 | Positive | 18.6 | Negative |
| **N308** | 0.435 | Positive | 35.1 | Negative |
| **N349** | 0.445 | Positive | 22.8 | Negative |

Nine samples were discordant at the DP238L-iELISA S/P cutoff of 0.30: five DP238L-positive/INgezim-negative and four DP238L-negative/INgezim-positive samples. No independent third-method adjudication was available.

## Table S3. Individual longitudinal S/P values for five experimentally infected pigs (0–21 dpi).

| Pig ID | 0 dpi | 3 dpi | 7 dpi | 10 dpi | 14 dpi | 21 dpi |
|---|---|---|---|---|---|---|
| Pig-01 | 0.013 | 0.006 | 0.320 | 0.426 | 0.742 | 1.187 |
| Pig-02 | -0.001 | 0.076 | 0.349 | 0.914 | 1.091 | 1.679 |
| Pig-03 | 0.004 | 0.008 | 0.269 | 0.402 | 0.553 | 0.764 |
| Pig-04 | -0.004 | -0.001 | 0.250 | 0.342 | 0.537 | 0.861 |
| Pig-05 | 0.001 | 0.070 | 0.376 | 0.570 | 0.917 | 1.266 |

The selected DP238L-iELISA cutoff was S/P = 0.30.

## Table S4. Individual results of the preliminary cross-reactivity panel.

| Target | Rep 1 | Rep 2 | Rep 3 | Mean S/P | SD | Distance from 0.30 cutoff |
|---|---|---|---|---|---|---|
| CSFV | 0.177 | 0.164 | 0.156 | 0.166 | 0.010 | 0.134 |
| PRV | 0.178 | 0.188 | 0.185 | 0.183 | 0.005 | 0.117 |
| PRRSV | 0.284 | 0.260 | 0.250 | 0.265 | 0.017 | 0.035 |
| PCV2 | 0.187 | 0.160 | 0.148 | 0.165 | 0.020 | 0.135 |
| JEV | 0.245 | 0.255 | 0.279 | 0.260 | 0.018 | 0.040 |
| CHO vector | 0.082 | 0.071 | 0.082 | 0.078 | 0.006 | 0.222 |

Each heterologous sample was tested in triplicate. Distance from cutoff = 0.30 − mean S/P. The panel was exploratory and was not designed to estimate population-level diagnostic specificity.
